# Single-molecule tracking reveals the dynamic turnover of Ipl1 at the kinetochores in *Saccharomyces cerevisiae*

**DOI:** 10.26508/lsa.202503290

**Published:** 2025-04-18

**Authors:** Nitesh Kumar Podh, Ayan Das, Akriti Kumari, Kirti Garg, Rashmi Yadav, Kirti Kashyap, Sahil Islam, Anupam Gupta, Gunjan Mehta

**Affiliations:** 1 Laboratory of Chromosome Dynamics and Gene Regulation, Department of Biotechnology, Indian Institute of Technology Hyderabad , Hyderabad, India; 2 School of Biomedical Sciences, University of New South Wales , Sydney, Australia; 3 Department of Physics, Indian Institute of Technology Hyderabad , Hyderabad, India

## Abstract

Aurora kinase B, Ipl1 in *Saccharomyces cerevisiae*, is a master regulator of cell division, required for checkpoint regulation, spindle assembly and disassembly, chromosome segregation, and cytokinesis. Decades of research employed ensemble averaging methods to understand its dynamics and function; however, the dynamic information was lost because of population-based averaging. Here, we use single-molecule imaging and tracking (SMIT) to quantify the recruitment dynamics of Ipl1 at the kinetochores and spindles in live cells. Our data suggest that Ipl1 is recruited to these locations with different dynamics. We have demonstrated how the recruitment dynamics of Ipl1 at the kinetochores during metaphase changes in the presence and absence of tension across the kinetochores, in the absence of protein phosphatase 1 (Glc7), and in the absence of its known recruiters (Ctf19 and Bub1). The SMIT of other chromosome passenger complex members (Bir1, Nbl1, Sli15) suggests their hierarchical assembly at the kinetochore. Hence, SMIT provides a dynamic view of the Ipl1 trafficking at the kinetochores and spindles.

## Abstract (precis)

Unlike ensemble averaging methods, this study visualizes the single molecules of Ipl1 in live yeast cells to quantify its recruitment dynamics and assembly as a part of the CPC at the kinetochores.

## Introduction

**Figure 1.**
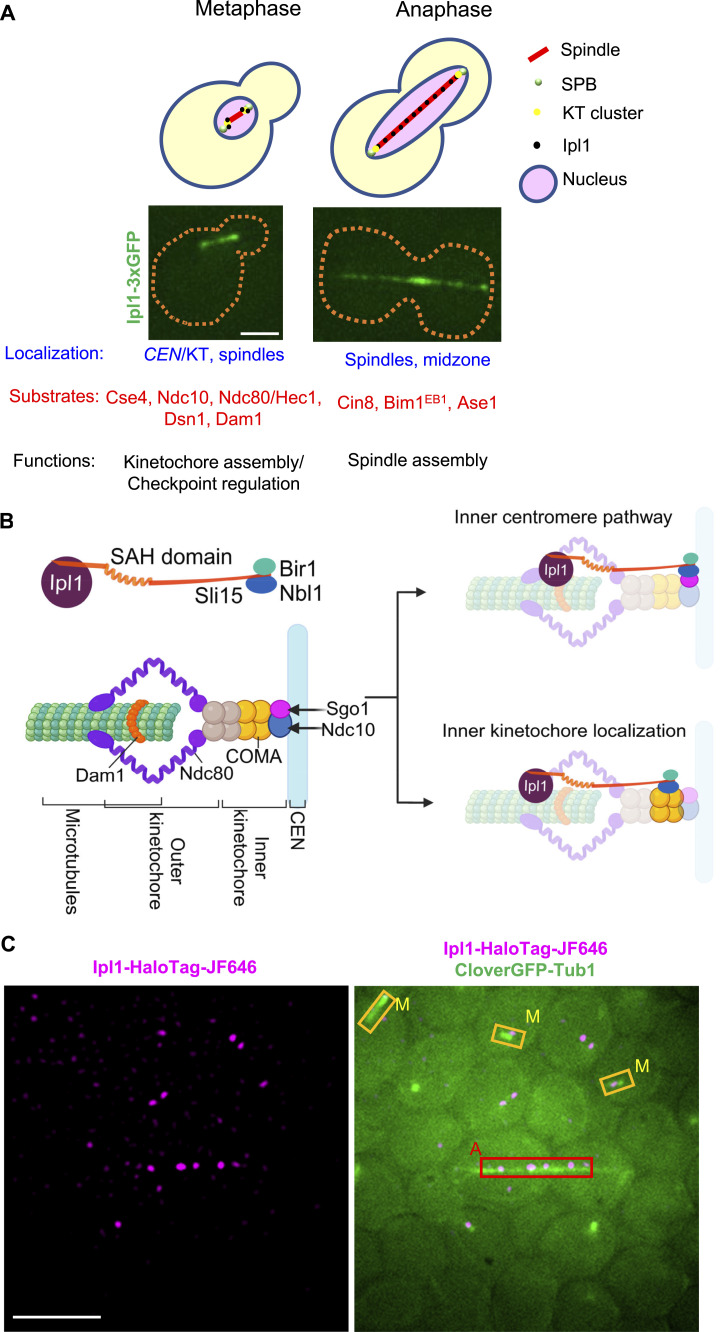
Introductory diagrams and the design of the experiment. **(A)** Schematic representation and corresponding microscopic images for the spatiotemporal localization of Ipl1 during mitosis. Ipl1 localizes to the kinetochores (during metaphase) and spindles (during anaphase). Its substrates and functions are depicted. Scale: 2 μm. **(B)** Mechanisms of CPC recruitment to the kinetochores: (Left) Composition of the kinetochore and CPC. (Right) Reported sites and pathways of CPC localization at the kinetochore. **(C)** Strategy for tracking single molecules of Ipl1-HaloTag-JF646 at different stages of mitosis at different locations. The left panel shows single molecules of Ipl1-HaloTag-JF646 (filtered image), and the right panel shows a merged image of CloverGFP-Tub1 with Ipl1-HaloTag-JF646. Based on spindle morphology, cells can be classified into metaphase and anaphase, and ROIs can be drawn to track the molecules within that area. Scale: 5 μm. *CEN*, centromere; KT, kinetochore.

Aurora kinases (AKs) play essential roles during cell division (mitosis and meiosis). They spatiotemporally phosphorylate various proteins at the kinetochores and spindles for functions such as kinetochore assembly, checkpoint regulation, spindle assembly/disassembly, chromosome segregation, and cytokinesis (Fig 1A) (Willems et al, 2018). The impaired function of AKs leads to severe chromosome missegregation, aneuploidy, and cancers. The human genome contains three genes encoding AKs: AK-A, AK-B, and AK-C. Their overlapping localization, functions, and substrates make studying specific functions/dynamics of AK-B in isolation challenging. In yeast *Saccharomyces cerevisiae*, Ipl1 (increase-in-ploidy) is a single essential AK, homologous to human AK-B, that serves as a valuable tool for studying its dynamics in isolation. Like human AK-B, Ipl1 localizes to the centromeres/kinetochores (during metaphase for kinetochore assembly and spindle checkpoint functions). It relocates to the spindles and spindle mid-zones (during anaphase and late anaphase for spindle assembly and disassembly, respectively, Fig 1A) (Biggins et al, 1999; Buvelot et al, 2003; Kotwaliwale et al, 2007). Ipl1 is recruited to the kinetochores or spindles as a part of the chromosome passenger complex (CPC, made up of Survivin [Bir1], Borealin [Nbl1], INCENP [Sli15], and AK B [Ipl1]). Sli15 links the Ipl1 and the Bir1-Nbl1 complex (Fig 1B). Two pathways are known to recruit the CPC to the kinetochores: (1) Bub1/Sgo1-mediated pathway that tethers the CPC to the inner kinetochore protein Ndc10 (Kawashima et al, 2010; Kelly et al, 2010; Tsukahara et al, 2010; Yamagishi et al, 2010), and (2) Sli15-mediated targeting of the CPC to the inner kinetochore protein Ctf19 (COMA complex) (Fischböck-Halwachs et al, 2019; García-Rodríguez et al, 2019). CPC is recruited to the spindle by the microtubule binding domain of Sli15 (Wheatley et al, 2001). Also, recent reports have demonstrated three discrete binding sites of the CPC: inner centromere, inner kinetochore, and spindles (Broad et al, 2020; Marsoner et al, 2022; Cairo et al, 2023). Conversely, two recent studies have demonstrated that the inner centromere and the inner kinetochore CPC targeting mechanisms are at least partially redundant for chromosome biorientation and cell viability in budding yeast (Fischböck-Halwachs et al, 2019; García-Rodríguez et al, 2019). In addition, mutations in the SAH domain of Sli15 prevent CPC localization to all three sites in budding yeast (Marsoner et al, 2022). All this evidence suggests that Ipl1 localization to all these three sites contributes to substrate phosphorylation at the outer kinetochore (Krenn & Musacchio, 2015; Broad et al, 2020).

Glc7 is a protein phosphatase 1 in yeast *S. cerevisiae*, and it dephosphorylates the Ipl1 substrates at the kinetochores (Pinsky et al, 2006). The interplay between Ipl1 and Glc7 still needs to be clearly understood to maintain the critical phosphorylation levels of Ipl1 substrates. Unlike the kinetochore/spindle-specific localization of Ipl1, Glc7 is localized in the entire nucleus throughout the cell cycle (Pinsky et al, 2006). However, its specific localization to the kinetochores/spindle-pole bodies is reported during anaphase (Bloecher & Tatchell, 2000; Pinsky et al, 2006).

Protein phosphorylation and dephosphorylation are dynamic processes in which a single protein molecule switches between ON and OFF states of phosphorylation because of the simultaneous presence of kinases and phosphatases in live cells (Pinsky et al, 2006; Gelens et al, 2018). Decades of research on AK-B/Ipl1 visualized and quantified its bulk localization in cells (using ChIP, immunofluorescence, Western blotting, and live-cell imaging) and quantified the phosphorylation level of its substrates after fixation and from the cell population. Hence, the dynamic behavior of individual molecules of Ipl1 and the dynamics of substrate phosphorylation were lost in the bulk measurements. The resulting static picture informs that Ipl1 localizes to several places and phosphorylates several substrates (Fig 1A). However, the dynamic information about Ipl1 action could not be quantified, such as how long Ipl1 binds to the centromeres/kinetochore and spindles for phosphorylating its substrates, how Ipl1 finds its target sites by exploring the entire nuclear space (target-search mechanism), how different modulators alter the recruitment dynamics of Ipl1 at the kinetochores/spindles.

In the last decade, single-molecule imaging and tracking (SMIT) has revolutionized how we understand biological processes (transcription, DNA replication, telomerase action) by revealing the dynamics of cellular processes at the single-molecule level (Izeddin et al, 2014; Ball et al, 2016; Schmidt et al, 2016; Mehta et al, 2018; Ranjan et al, 2020; Nguyen et al, 2021; Podh et al, 2021). However, the dynamic regulation of any kinase/phosphatase is not thoroughly quantified despite their dynamic interplay to regulate various biological signaling cascades. In this report, we use the SMIT method to quantify the fast dynamics of Ipl1 in live yeast cells. Ipl1 shows fast exchange/turnover at the kinetochores compared with the spindles, suggesting the importance of dynamic turnover in regulating kinetochore function (kinetochore assembly/checkpoint regulation). Upon depleting Glc7 during metaphase, the residence time and the fraction of specific bound molecules of Ipl1 increase at the kinetochores, suggesting that Glc7 is required for the fast exchange of Ipl1 at the kinetochores. Ipl1 is best known for its role in tension sensing at the kinetochores during metaphase for error correction. We quantified the dynamics of Ipl1 at the kinetochores during metaphase in the presence and absence of tension. Our data suggest that once the biorientation is achieved and the kinetochores are under tension, Ipl1 diminishes its binding at the kinetochores. Upon releasing the tension by depolymerizing microtubules, Ipl1 relocates to the kinetochores. Hence, tension across the kinetochores keeps the Ipl1 away, whereas reduced tension, irrespective of erroneous attachment, recruits Ipl1 to the kinetochores. We tracked the dynamics of Ipl1 in the absence of Ctf19 or Bub1, known recruiters of Ipl1 at the kinetochores. Our data suggest that the absence of Ctf19 reduces the specific bound fraction of Ipl1 at the kinetochore and increases the search time of Ipl1 to find the kinetochores, whereas the absence of Bub1 abolishes the specific binding of Ipl1 to the kinetochore. SMIT of the other members of the CPC suggests a hierarchical assembly of them at the kinetochores during metaphase based on their residence times. Nbl1-Bir1 assembles first at the kinetochores, followed by the Sli15-Ipl1 complex. In summary, this study reveals the dynamics of Ipl1 recruitment and its target-search mechanism to achieve its multifaceted functions during cell division.

## Results

### Strategy for SMIT of Ipl1 during different stages of mitosis

**Figure S1.**
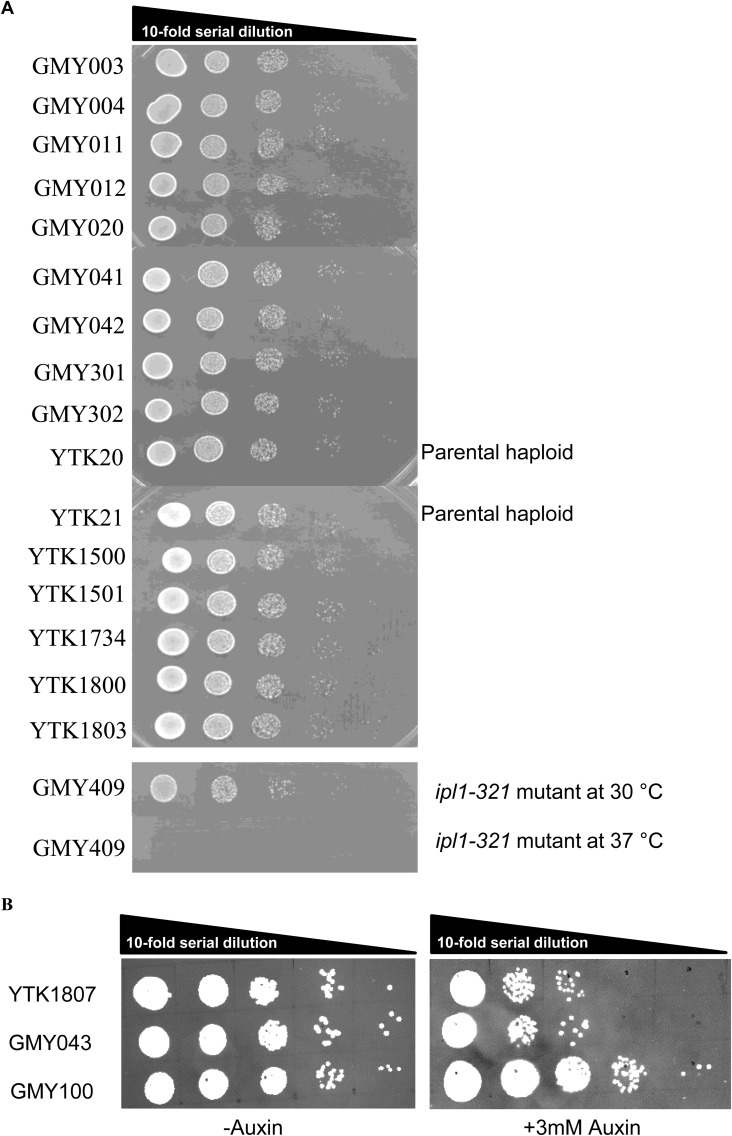
Spot tests to check the cell viability for all the C-terminal fusions and deletions used in this research. **(A)** 10-fold serial dilutions of all the haploid strains used in this study were plated on YPD plates and incubated at 30°C for 48 h, except otherwise stated. **(B)** Functional assay for auxin-inducible depletion of the Cdc20-AID* and Glc7-AID*. 10-fold serial dilutions were plated on YPD and YPD+3-mM auxin plates and incubated at 30°C for 48 h. GMY100 was used as a parental strain (without any genetic engineering).

For visualizing single molecules of Ipl1 in live cells of *S. cerevisiae*, we endogenously fused a protein self-labeling enzyme tag HaloTag to the C terminus of the *IPL1* gene for the controlled labeling of Ipl1. To identify the stages of the cell cycle and to mark the position of Ipl1 localization (kinetochores during metaphase, spindles during anaphase, Fig 1A), we expressed CloverGFP-Tub1. We used diploid strains for all the experiments with homozygous *pdr5*Δ, heterozygous *IPL1-HaloTag*, and heterozygous *CloverGFP-TUB1*. To check the functionality of the protein fusions and the overall viability of the genetically engineered strains, spot tests were performed with a 10-fold serial dilution on YPD plates (Fig S1A). None of the strains showed significant growth delay compared with the parental strain. This result suggests that the protein fusions are functional and cells are healthy.

**Figure S2.**
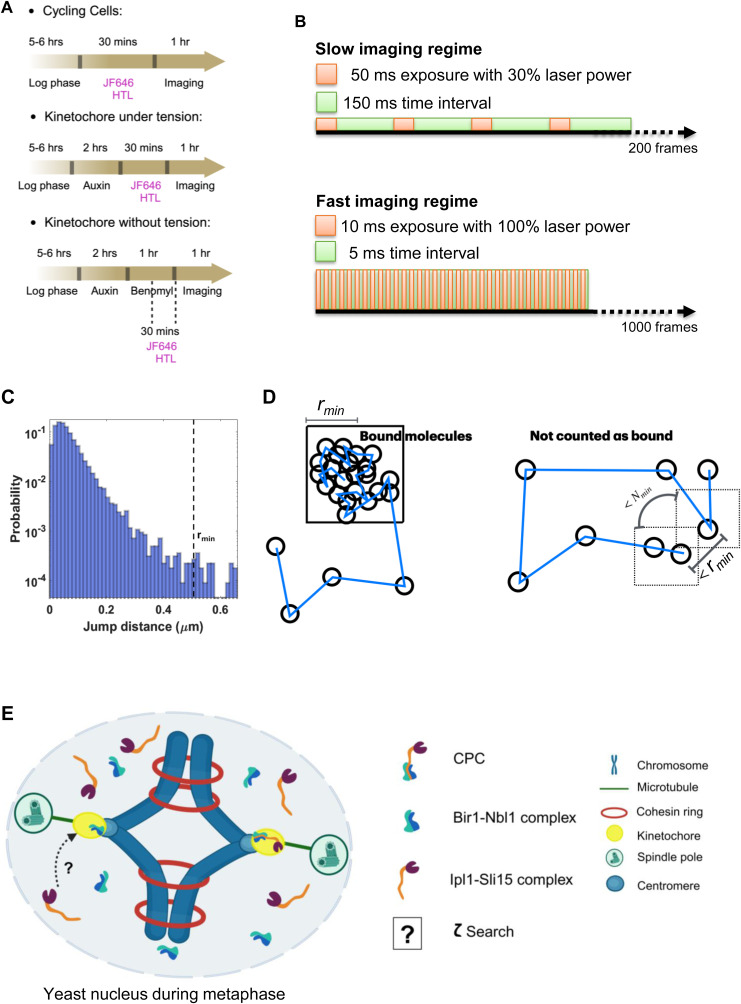
Culturing, imaging, and tracking strategies. **(A)** Culturing regime: overnight grown cells were inoculated in a fresh CSM media and grown for 5 h (for log phase). Cells were treated with JF646-HTL for 30 min, washed twice with CSM, and imaged under the microscope. When necessary, auxin treatment was given 2 h before the addition of JF646-HTL. When necessary, benomyl treatment was given for 1 h after auxin treatment, along with incubation with JF646-HTL for 30 min. In all cases, the cells were imaged for 1 h from the same agarose pad. Every 1 h, fresh samples were taken. **(B)** Imaging regimes: time-lapse videos were acquired with a “slow-imaging regime” (200-ms time interval for 200 frames, 50-ms exposure time, 30% laser power) to estimate dwell time, whereas time-lapse videos were acquired with a “fast-imaging regime” (15-ms interval for 1,000 frames, 10-ms exposure time, 5-ms camera processing time, 100% laser power) to estimate diffusion parameters (fraction of bound and unbound molecules, and diffusion coefficients). **(C)** SMIT was performed for kinetochore protein Ndc10-3xGFP foci to define a benchmark for the kinetochore-bound protein. Frame-to-frame displacements were plotted as a histogram. The *r*_*min*_ value defines the maximum frame-to-frame distance traveled by 99% of the foci. **(D)** Schematics to explain the terms: *r*_*min*_ and *N*_*min.*_ *r*_*min*_ is the maximum frame-to-frame distance traveled by 99% of Ndc10-3xGFP foci in time-lapse videos acquired with a slow-imaging regime. *N*_*min*_ is the minimum number of frames for which a bound molecule should not travel more than *r*_*min*_ distance frame to frame. **(E)** Schematics of the target-search mechanism of CPC to find kinetochores during metaphase. τ_search_ is the time required by Ipl1 to reach the kinetochore.

For sparse labeling of Ipl1-HaloTag, the log phase cells were treated with 30 nM JF646-HTL for 30 min and observed under the single-molecule imaging microscope (Fig S2A, Materials and methods). To quantify a broad range of kinetic behavior of Ipl1, time-lapse videos were acquired with two imaging regimes: “fast regime” and “slow regime” (Fig S2B, Materials and methods) (Podh et al, 2022).

Based on the CloverGFP-Tub1 morphology, the stages of mitosis (metaphase and anaphase) can be identified and the regions of interest (ROIs) can be defined for tracking the single molecules of Ipl1-HaloTag-JF646 at kinetochores and spindles (Fig 1C). Single-molecule tracking and data analysis were performed as described in the Materials and Methods section (Fig S2) (Podh et al, 2022).

### Ipl1 is recruited to the kinetochores and spindles with different dynamics

For SMIT of Ipl1 at metaphase and anaphase, we used a diploid yeast strain with homozygous *pdr5*Δ, heterozygous *IPL1-HaloTag*, and heterozygous *CloverGFP-TUB1* (YTK1804). The diploid strain was grown to the mid-log phase, the Ipl1-HaloTag was labeled with 30 nM of JF646-HTL for 30 min, and cells were imaged under the single-molecule imaging microscope using a slow-imaging regime (Materials and methods, Fig S2A and B, Video 1). The imaging and tracking were performed as described in the Materials and Methods section.

Video 1 Representative time-lapse video (acquired using a slow-imaging regime) for single-molecule tracking of Ipl1-HaloTag-JF646 over the kinetochores and spindles. Play speed: 1x. Download video

**Figure 2.**
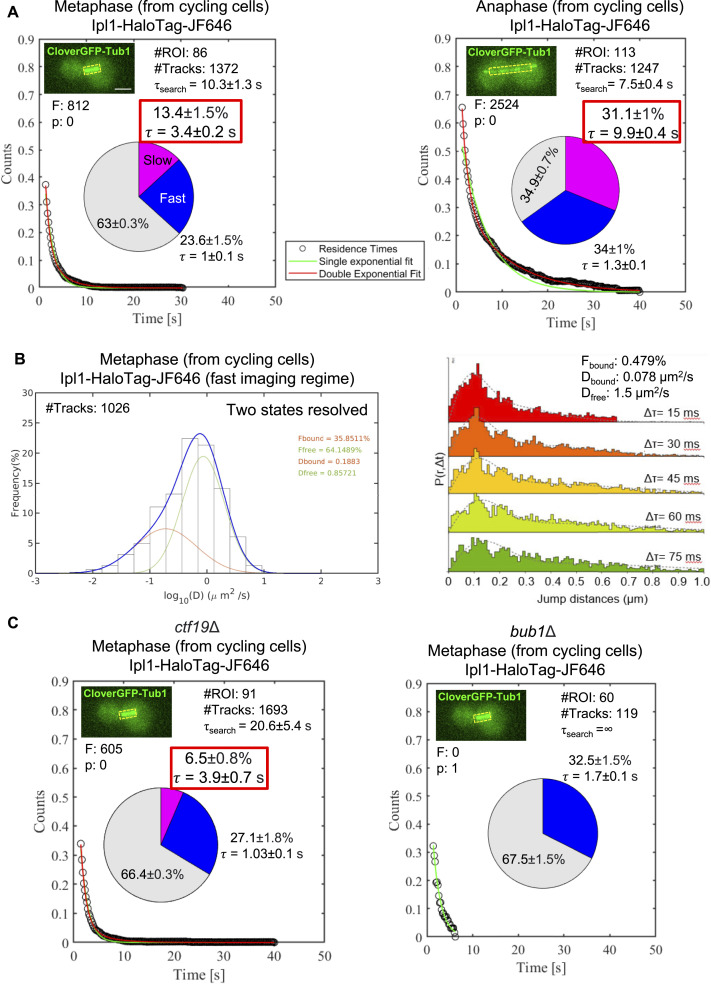
SMIT of Ipl1-HaloTag-JF646 at different stages of mitosis and in the absence of Ctf19 and Bub1. **(A)** Residence time analysis of Ipl1 during metaphase and anaphase using a slow-imaging regime. Survival probability distributions were fitted with single (green)- or double (red)-exponential decay curves. For both stages, the survival distribution fitted best to the double-exponential decay, suggesting two populations of residence times. **(B)** SMIT of Ipl1-HaloTag-JF646 at metaphase kinetochores using a fast-imaging regime. A histogram of LogD was used to identify the number of diffusion states of the molecules. Spot-On–based kinetic modeling was used for the robust quantification of the fraction of bound (F_bound_) molecules and its mean D value (D_bound_). **(C)** SMIT of Ipl1-HaloTag-JF646 in the absence of Ctf19 and Bub1 during metaphase using a slow-imaging regime. **(A)** Survival probability distributions and pie charts are represented as (A). For (A, C), the pie charts represent the fraction of molecules bound with long residence time (pink fraction) and the fraction of molecules bound with short residence time (blue fraction), along with their mean residence times (τ). The gray fraction represents diffusing molecules. “#Tracks” represents the total number of tracks analyzed, “#ROIs” represents the total number of cells tracked, “τ_search_” represents the target-search time, “F” represents the value of the “*F* test” to distinguish between single- versus double-exponential fitting, and “P” represents the *P*-value of the *F* test. The inset images show the ROIs used for tracking. Scale: 2 μm. Source data are available for this figure.

**Figure S3.**
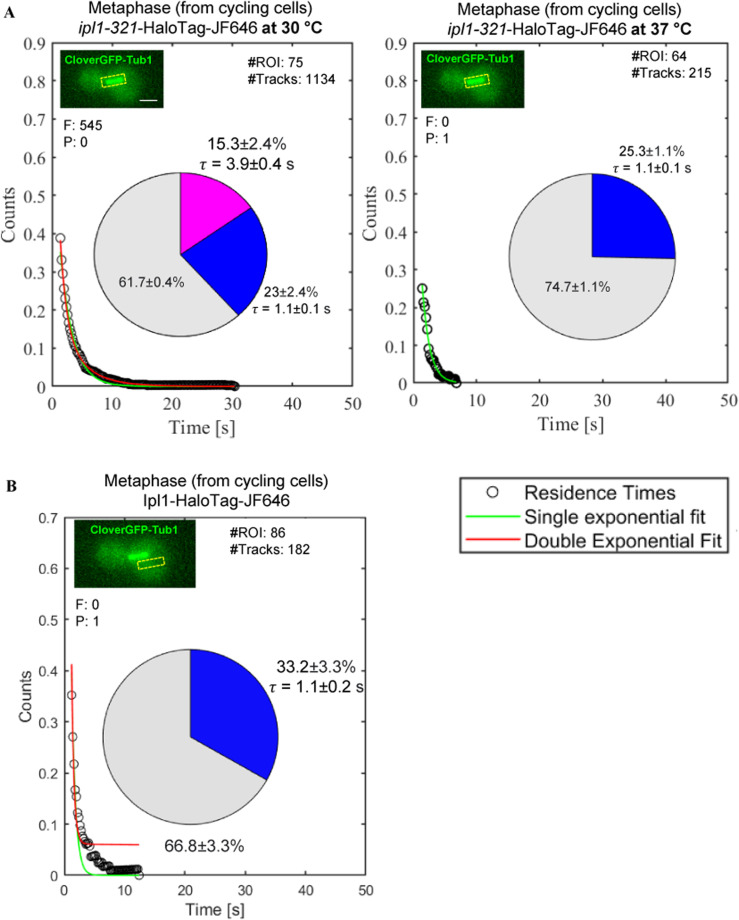
Experiments to ensure that the slow fraction (pink fraction in the pie charts) represents specific bound molecules of Ipl1 at the kinetochores that are responsible for phosphorylation. **(A)** Residence time analysis of *ipl1-321*-HaloTag temperature-sensitive mutant at the permissive (30°C) and restrictive (37°C) temperatures. **(B)** From the same set of videos used for tracking Ipl1-HaloTag-JF646 at metaphase (for Fig 2A), molecules were tracked by making ROIs away from the kinetochores and spindles (at nonspecific sites). For (A, B), survival probability distributions were fitted with single (green)- or double (red)-exponential decay curves. The pie charts represent the fraction of molecules bound with long residence time (pink fraction) and the fraction of molecules bound with short residence time (blue fraction), along with their mean residence times (τ). The gray fraction represents diffusing molecules. “#Tracks” represents the total number of tracks analyzed, “#ROIs” represents the total number of cells tracked, “F” represents the value of the “*F* test” to distinguish between single- versus double-exponential fitting, and “P” represents the *P*-value of the *F* test. The inset images show the ROIs (yellow) used for tracking. Scale: 2 μm. Source data are available for this figure.

We observed that the survival probability distributions for both stages fit well to the double-exponential decay (Fig 2A, red line), suggesting two types of binding events: (1) binding with long residence time (slow fraction, pink fraction in the pie chart) and (2) binding with short residence time (fast fraction, blue fraction in the pie chart). To ensure which fraction represents the Ipl1 molecules specifically bound to the target sites (kinetochores) for phosphorylation, we performed the same analysis for the temperature-sensitive *ipl1-321* mutant (Fig S3A). This mutant is known for its reduced/negligible kinase activity at a restrictive temperature (37°C) (Biggins et al, 1999; Buvelot et al, 2003). We observed that the survival probability distribution fits well to the double-exponential decay at the permissive temperature (30°C; Fig S3A, left panel); however, at the restrictive temperature (37°C), we found only a few tracks (215) at the kinetochores and the survival probability distribution fits to single-exponential decay (Fig S3A, right panel). The *ipl1-321* mutant failed to show specific binding (pink fraction) at the kinetochores at 37°C. To further validate that the pink fraction only represents the specific binding, we tracked Ipl1-HaloTag-JF646 (from the same videos acquired for Fig 2A, left panel) at nonspecific sites (away from its actual binding sites, kinetochores and spindles, Fig S3B). We found only a few tracks (182), and the survival distribution fits well to the single-exponential decay, suggesting the absence of specific binding (pink fraction). These experiments confirmed that the pink fraction represents specifically bound molecules of Ipl1 responsible for the phosphorylation of its substrates, whereas the blue fraction represents nonspecifically/transiently bound molecules of Ipl1. Henceforth, we will discuss the specific bound fraction (pink fraction) only, as it represents Ipl1 molecules involved in substrate phosphorylation. In contrast, the blue and gray fractions do not contribute directly toward the Ipl1 function.

We tracked Ipl1-HaloTag-JF646 at the kinetochores (during metaphase) and spindles (during anaphase, Fig 2A). Ipl1 showed residence times of 3.4 ± 0.2 s and 9.9 ± 0.4 s, respectively (pie charts, pink fractions, *P* < 0.001). Also, the specific bound fraction (pink fraction) changes significantly between metaphase and anaphase (13.4 ± 1.5% and 31.1 ± 1%, respectively, *P* < 0.001). We also calculated the target-search time (τ_search_), as described in the Materials and Methods section. The target-search time is defined as the time required by Ipl1 to reach its target sites (kinetochores or spindle, Fig S2E). We found that the search time for Ipl1 to find kinetochores during metaphase is 10.3 ± 1.3 s, whereas it takes 7.5 ± 0.4 s to reach the spindles during anaphase. These results suggest that Ipl1 is recruited to the kinetochores and spindles with different dynamics for phosphorylation.

Several reports have shown three discrete binding sites (inner centromere, inner kinetochore, and outer kinetochore) of AK-B/Ipl1 during metaphase (Bloecher & Tatchell, 2000; Broad et al, 2020; Marsoner et al, 2022). While tracking Ipl1 at the kinetochores during metaphase, we could not differentiate the locations of these three sites because of the diffraction-limited resolution of the microscope. Hence, our metaphase tracking data represent the molecules at all three sites. To address this conundrum, we quantified the diffusion coefficients (D) of Ipl1 populations at the kinetochores during metaphase in cycling cells (Fig 2B) using the fast-imaging regime (Materials and methods) and Spot-On–based kinetic modeling (Hansen et al, 2018). The rationale is that the Ipl1 populations at these three discrete binding sites may have different mean D values. The diffusion coefficient analysis performed here (mean-squared displacement [MSD]–based logD histograms to identify the resolvable states [e.g., 2 states or 3 states] within the entire population) can resolve up to three different D values (for bound, intermediate bound, and free molecules), as reported previously for the transcription pre-initiation complex components TBP and TFIIE (Nguyen et al, 2021). We observed only two populations of Ipl1 (bound and unbound) at the kinetochores based on this analysis (Fig 2B, left panel). This approach can produce unreliable quantification for the mean D value because it depends on the choice of track length and linear fits to MSD. Therefore, the Spot-On–based kinetic modeling (Hansen et al, 2018) was used for the robust quantification of the mean D values. This analysis showed a 48% bound fraction with a mean D value of 0.08 μm^2^/s (Fig 2B, right panel). This result suggests that despite having multiple discrete sites for the localization of Ipl1 at the kinetochores, all the molecules of Ipl1 at the kinetochores have the same diffusion characteristics. To support this result, two recent studies have demonstrated that the inner centromere and the inner kinetochore CPC targeting mechanisms are at least partially redundant for chromosome biorientation and cell viability in budding yeast (Fischböck-Halwachs et al, 2019; García-Rodríguez et al, 2019). In addition, mutations in the SAH domain of Sli15 prevent CPC localization to all three sites in budding yeast (Marsoner et al, 2022). All this evidence suggests that despite having three discrete sites for Ipl1 localization at the kinetochores, Ipl1 binds to these sites with the same diffusion characteristics that may indicate functional redundancy.

### The absence of Ctf19 or Bub1 reduces the specific bound fraction of Ipl1 at the kinetochores and increases the target-search time

As mentioned in the introduction, Ipl1 is recruited to the kinetochore as a part of the CPC by two pathways: (A) Bub1/Sgo1-mediated recruitment and (B) Ctf19 (COMA complex)-mediated recruitment (Fig 1B). Both these mechanisms work redundantly to promote chromosome biorientation. Hence, we want to address how the absence of any of these mechanisms affects the recruitment dynamics of Ipl1 at the kinetochore. We created homozygous deletions of *BUB1* or *CTF19* in the diploid strain used above (GMY009, GMY303). The cells were labeled and imaged for SMIT, and the data analysis was performed as described before (Fig 2A).

We observed that the absence of Ctf19 reduces the specific bound fraction of Ipl1 at the kinetochore significantly (from 13.4 ± 1.5% [Fig 2A] to 6.5 ± 0.8%, *P* < 0.001 [Fig 2C, left panel]), without altering its residence time significantly (from 3.4 ± 0.2 s [Fig 2A] to 3.9 ± 0.7 s [Fig 2C], *P* < 0.05). Conversely, the absence of Bub1 completely abolished the specific bound fraction of Ipl1 at the kinetochore (Fig 2C, right panel), suggesting the dominance of the Bub1-mediated recruitment of Ipl1 at the kinetochores over the Ctf19-mediated recruitment. Both these pathways are not entirely independent. Without Bub1, the Ctf19-mediated pathway cannot recruit Ipl1 to the kinetochores.

As Ctf19 and Bub1 are the known recruiters of Ipl1 to the kinetochores, we asked if Ipl1 takes a long time to find the kinetochores in their absence. We calculated the target-search time (τ_search_). The absence of Ctf19 increases the target-search time of Ipl1 to find the kinetochores, from 10.3 ± 1.3 s (Fig 2A, metaphase) to 20.6 ± 5.4 s (Fig 2C, *ctf19*Δ, *P* < 0.001), whereas the target-search time in the absence of Bub1 was found to be infinite (Fig 2C, *bub1*Δ). This result suggests that Ipl1 takes a long time to find the kinetochores in the absence of Ctf19, whereas it fails to reach the kinetochores in the absence of Bub1.

### Tension across the kinetochores diminishes Ipl1 binding to the kinetochores

**Figure 3.**
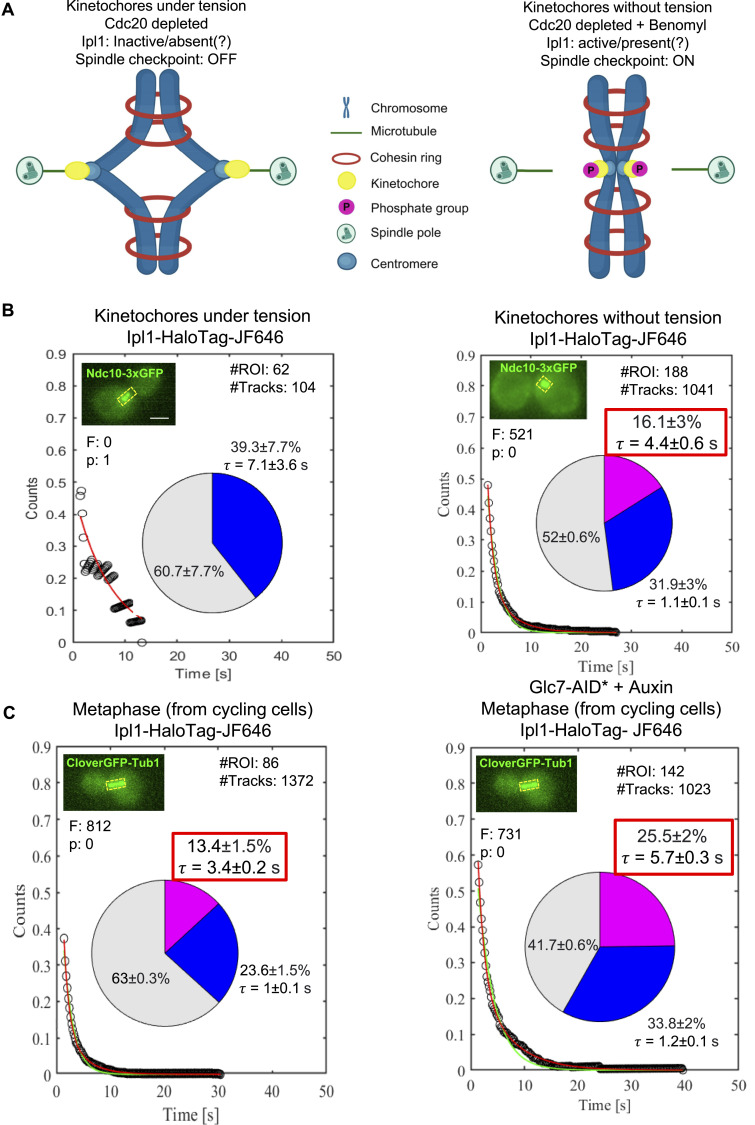
Effect of tension across the kinetochores and the absence of Glc7 on the dynamics of Ipl1 at metaphase. **(A)** Schematic representation of the simulated experimental conditions for keeping the kinetochores under tension and releasing the tension. **(B)** SMIT of Ipl1-HaloTag-JF646 in the presence and absence of tension at the kinetochores during metaphase. Survival probability distributions and pie charts are represented as Fig 2A. **(C)** SMIT of Ipl1-HaloTag-JF646 in the presence and absence of Glc7 at metaphase. Survival probability distributions and pie charts are represented as Fig 2A. The data shown on the left panel are the same as it is shown in Fig 2A (for metaphase); however, it has been shown again for easy comparison with the absence of Glc7. For (B, C), the pie charts represent the fraction of molecules bound with long residence time (pink fraction) and the fraction of molecules bound with short residence time (blue fraction), along with their mean residence times (τ). The gray fraction represents diffusing molecules. “#Tracks” represents the total number of tracks analyzed, “#ROIs” represents the total number of cells tracked, “τ_search_” represents the target-search time, “F” represents the value of the “*F* test” to distinguish between single- versus double-exponential fitting, and “P” represents the *P*-value of the *F* test. The inset images show the ROIs used for tracking. Scale: 2 μm. Source data are available for this figure.

**Figure S4.**
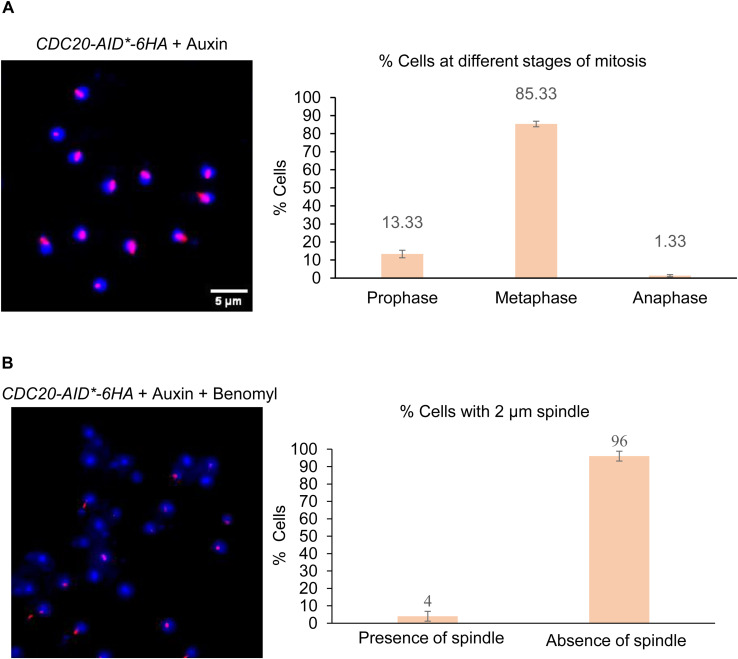
Immunofluorescence to validate metaphase arrest and microtubule depolymerization. **(A)** Immunofluorescence was performed using anti-tubulin antibodies and DAPI staining to determine the stages of the cell cycle. After 2.5 h of auxin treatment (for Cdc20 depletion), >85% of cells showed 2 μm long tubulin staining indicating the metaphase stage. **(B)** Upon microtubule depolymerization (by adding benomyl for 1 h), loss of 2 μm long tubulin structures was observed in >95% of cells, confirming microtubule depolymerization. The single foci in some of the nuclei represent SPBs, where the monomers of tubulin are present.

**Figure S5.**
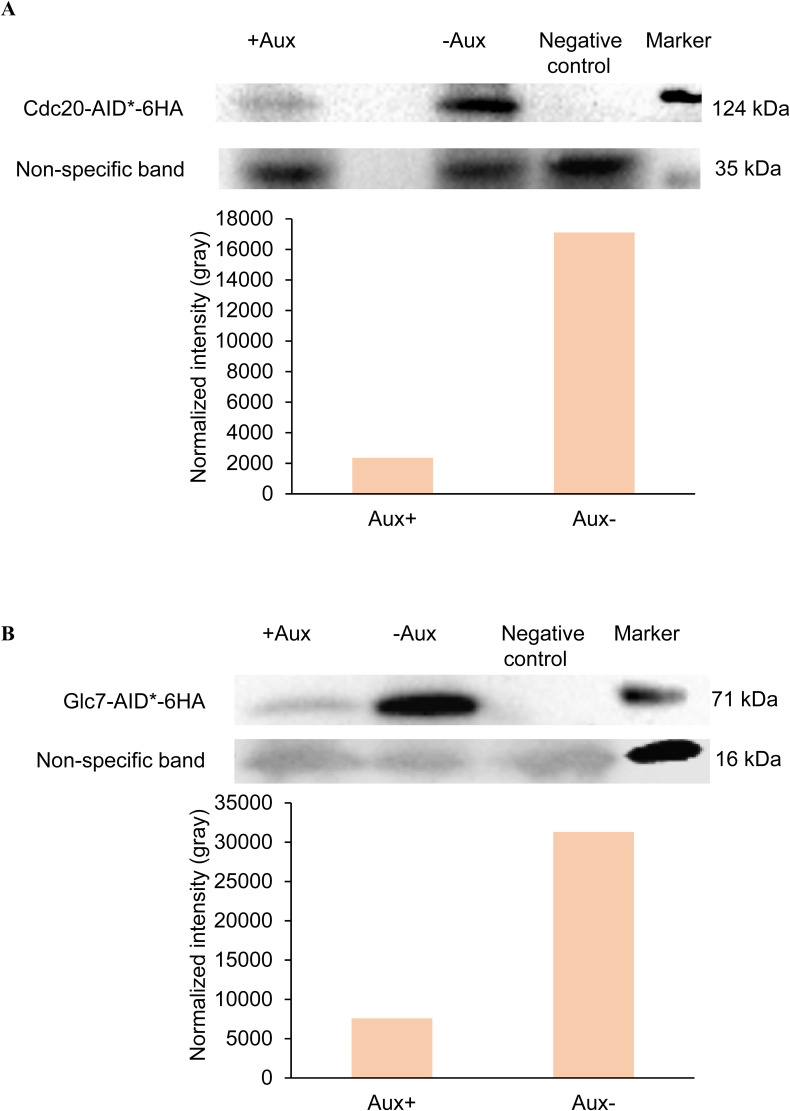
Western blotting to confirm the auxin-induced depletion of Glc7 and Cdc20. **(A, B)** Addition of auxin depleted Glc7 and Cdc20 significantly (>4 times for Glc7 and >8 times for Cdc20). A nonspecific band on the Western blot was used as a loading control. Source data are available for this figure.

Ipl1 is best known for its role in tension sensing at the kinetochores during metaphase for error correction (Biggins & Murray, 2001; McVey et al, 2021; Edgerton et al, 2023). So, we asked how the tension across the kinetochore alters the dynamic recruitment of Ipl1 during metaphase. We designed an experimental strategy by which we can keep all the kinetochores under tension, followed by adding a microtubule depolymerizing drug (benomyl) to release the tension. We used an auxin-inducible degron system (Materials and methods) for the conditional depletion of the activator of the anaphase-promoting complex, Cdc20. By depleting Cdc20, cells were arrested at metaphase with all the kinetochores attached to the spindles, and they were under tension because of the pulling forces exerted by the spindles (Fig 3A). For this purpose, we genetically engineered a diploid yeast strain with homozygous *pdr5*Δ, homozygous CDC20-AID*-6HA, heterozygous *IPL1-HaloTag*, heterozygous *NDC10-GFP*, and heterozygous *pADH-AFB2* (YTK1807). For this experiment, we used Ndc10-GFP instead of CloverGFP-Tub1 because microtubule depolymerization may lead to the disappearance of CloverGFP-Tub1, which may increase inaccuracies in ROI generation for tracking. Ndc10 is an inner kinetochore protein that directly visualizes the position of the metaphase kinetochores in the presence and absence of microtubules. It also facilitates ROI generation for tracking. SMIT was performed for Ipl1-HaloTag-JF646 after adding auxin for 2 h to the log phase cells (Fig S2A and B, Video 2). We added benomyl at 90 μg/ml concentration to release the tension and incubated the cells for an additional 1 h (Fig S2A). We performed immunofluorescence analysis using anti-tubulin antibodies and DAPI from both these samples to confirm the metaphase arrest upon depleting Cdc20 (Fig S4A) and microtubule depolymerization upon benomyl treatment (Fig S4B). Immunofluorescence analysis confirmed that >85% of cells showed metaphase spindle upon depleting Cdc20 and the absence of elongated microtubules in >95% of cells after treatment with benomyl. Western blotting was performed to confirm the conditional depletion of Cdc20-AID*-6HA (Fig S5A).

Video 2 Representative time-lapse video (acquired using a slow-imaging regime) for single-molecule tracking of Ipl1-HaloTag-JF646 over the kinetochores that are under tension. Play speed: 1x. Download video

Our SMIT data demonstrated that the dynamic recruitment of Ipl1 diminished at the kinetochores when the kinetochores were under tension (Fig 3B, Video 2), as seen by the absence of the specifically bound fraction of Ipl1. Upon releasing the tension, Ipl1 relocalized to the kinetochores (16.1 ± 3% pink fraction with a residence time of 4.4 ± 0.6 s, Fig 3B). This result aligns with a recent report in which authors have demonstrated the same phenomenon using Ipl1-3xGFP (Edgerton et al, 2023). These data suggest that the tension across the kinetochores can modulate the dynamic recruitment of Ipl1 at the kinetochore.

### Conditional depletion of Glc7 increases the molecular crowding of Ipl1 at the metaphase kinetochores

Glc7 (protein phosphatase 1) antagonizes Ipl1-mediated phosphorylation (Pinsky et al, 2006). Ipl1 and Glc7 modulate the phosphorylation of a common set of substrates (Ndc10, Dam1, H3). However, the precise interplay between the kinase and phosphatase needs to be better understood. To address this question, we quantified the single-molecule dynamics of Ipl1 at the kinetochore in the absence of Glc7 during metaphase. We genetically engineered a diploid yeast strain with homozygous *pdr5*Δ, homozygous GLC7-AID*-6HA, heterozygous *IPL1-HaloTag*, heterozygous *CloverGFP-TUB1*, and heterozygous *pADH-AFB2* (GMY043). SMIT was performed for Ipl1-HaloTag-JF646 with and without auxin treatment for 2 h (Fig S2A). The conditional depletion of Glc7-AID*-6HA was confirmed by Western blotting (Fig S5B).

We observed that in the absence of Glc7, the fraction of specific bound molecules of Ipl1-HaloTag-JF646 increases from 13.4 ± 1.5% to 25.5 ± 2% (*P* < 0.001, Fig 3C) and the residence time increases from 3.4 ± 0.2 s to 5.7 ± 0.3 s (*P* < 0.001, Fig 3C). This result suggests that the absence of Glc7 increases the retention of Ipl1 at the kinetochores. Hence, the presence of Glc7 is required for the fast turnover/exchange of the Ipl1 at the kinetochores during metaphase. Rapid turnover of Ipl1 at the metaphase kinetochores may keep Glc7 away from their common substrates to keep them phosphorylated.

### SMIT of CPC components reveals the hierarchical assembly of the CPC at the kinetochores during metaphase

Ipl1 is recruited to the kinetochores during metaphase as a part of the CPC (Carmena et al, 2012). It is unknown how this four-membered protein complex assembles at the kinetochores, whether a preassembled complex comes to the kinetochore, or whether it is assembled at the kinetochore by bringing one after another member. To address this question, we designed an experiment to track the single-molecule dynamics of other members of the CPC (Bir1, Nbl1, Sli15). The hypothesis is that if the entire complex is recruited to the kinetochores as a preassembled complex, all components' residence time will be comparable. Otherwise, if there is a hierarchical assembly, all these components will show different residence times, depending on their order in the assembly process. The component recruited first will show the longest residence time, compared with the component recruited last. For this experiment, we engineered four different strains with heterozygous CloverGFP-Tub1, homozygous *pdr5*Δ, and heterozygous C-terminal HaloTag fusions to the CPC components (Bir1, Nbl1, Sli15, and Ipl1; GMY013, GMY014, GMY044, and GMY1804, respectively). We confirmed the functionality of the C-terminal fusions by the spot test (Fig S1A) and could not observe significant growth defects.

**Figure 4.**
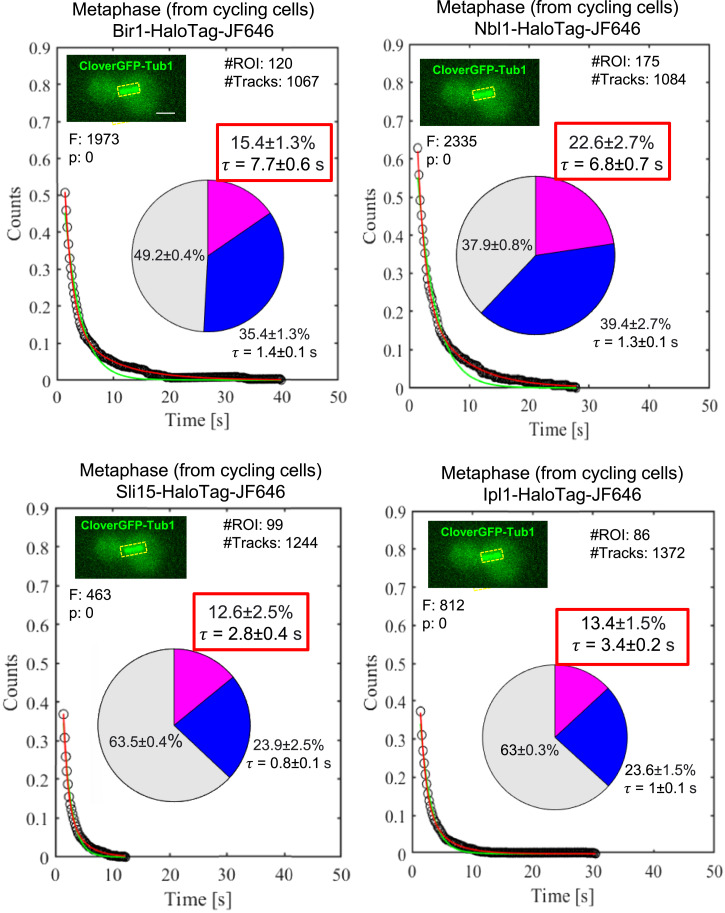
Hierarchical assembly of the CPC at the kinetochore revealed by SMIT of Bir1, Nbl1, Sli15, and Ipl1 at metaphase. SMIT of Bir1-HaloTag-JF646, Nbl1-HaloTag-JF646, Sli15-HaloTag-JF646, and Ipl1-HaloTag-JF646 during metaphase using a slow-imaging regime. Survival probability distributions and pie charts are represented as Fig 2A. The pie charts represent the fraction of molecules bound with long residence time (pink fraction) and the fraction of molecules bound with short residence time (blue fraction), along with their mean residence times (τ). The gray fraction represents diffusing molecules. “#Tracks” represents the total number of tracks analyzed, “#ROIs” represents the total number of cells tracked, “τ_search_” represents the target-search time, “F” represents the value of the “*F* test” to distinguish between single- versus double-exponential fitting, and “P” represents the *P*-value of the *F* test. The inset images show the ROIs used for tracking. Scale: 2 μm. The data shown for Ipl1-HaloTag-JF646 are the same as it is shown in Fig 2A (for metaphase). However, it has been shown again for easy comparison with the other CPC subunits. Source data are available for this figure.

SMIT was performed for all four diploid strains, as mentioned above. We observed that the residence times of Bir1 and Nbl1 are comparable (7.7 ± 0.6 s and 6.8 ± 0.7 s, respectively, *P* = not significant, Fig 4), and those of Sli15 and Ipl1 are comparable (2.8 ± 0.4 s and 3.4 ± 0.2 s, respectively, *P* < 0.05, Fig 4). However, there is a significant difference in the residence time of the Bir1-Nbl1 complex and the Sli15-Ipl1 complex (*P* < 0.001). These results suggest that Bir1-Nbl1 and Sli15-Ipl1 are recruited to the kinetochores as separate heterodimeric complexes. It reveals the possible hierarchical assembly of the CPC components to the kinetochores during metaphase; Bir1-Nbl1 heterodimer is recruited first to the kinetochore, followed by Sli15-Ipl1 heterodimer.

## Discussion

AK-B in humans and Ipl1 in yeast *S. cerevisiae* have been studied for a few decades as they are the most promising targets for cancer therapies (Lakkaniga et al, 2024). Throughout these years, several ensemble averaging methods such as ChIP, immunofluorescence, live-cell imaging, Western blotting, and proteomic analysis have been employed to understand the dynamics and functions of AK-B/Ipl1. However, all these methods provided a static picture of AK-B/Ipl1 localization and function because of population-based averaging. The dynamic information was lost. SMIT has emerged as a powerful technique to visualize and quantify the dynamics of proteins in live cells with high spatiotemporal resolution (Podh et al, 2021). Here, we employed this method to quantify the recruitment dynamics of Ipl1 in yeast *S. cerevisiae* and to understand how it changes in the absence of several modulators.

### Novel insights from the SMIT of Ipl1

Using this method, we could quantify the timescale of Ipl1 activities, such as the residence time of Ipl1 at the kinetochores (e.g., 3.4 ± 0.2 s at metaphase, 9.9 ± 0.4 s at anaphase), how long it takes to find its target sites (kinetochores) during metaphase (e.g., 10.3 ± 1.3 s in WT, 20.6 ± 5.4 s in the absence of Ctf19), and what fraction of molecules are involved in phosphorylation (e.g., 13.4 ± 1.5% during metaphase, 31.1 ± 1% during anaphase). Intriguingly, Ipl1 spends less time at the kinetochores (3.4 ± 0.2 s) compared with spindles (9.9 ± 0.4 s), though it has more substrates to phosphorylate at the kinetochores (Cse4, Ndc10, Ndc80/Hec1, Dam1, Dsn1) compared with spindles (Cin8, Bim1, Ase1). Hence, the rapid turnover at the kinetochore may be necessary for its kinetochore functions (kinetochore assembly and spindle checkpoint). It may create a molecular crowd around kinetochores that keeps the phosphatase (Glc7) away, so the substrates of Ipl1 at the kinetochore remain phosphorylated. A recent report from the Tanaka Lab shows that rapid turnover of Ipl1 at the kinetochore is not required to achieve biorientation (Li et al, 2023). These experiments were done in metaphase-arrested cells (by depleting Cdc20) and using artificial tethering of Ipl1-Sli15 to the kinetochore proteins Mif2 and Ndc80. So, it is possible that in cycling cells, rapid turnover of Ipl1 at the kinetochore is essential to keep the Glc7 away to support other functions such as kinetochore assembly and spindle checkpoint. Our data also support this hypothesis, as in the absence of Glc7, the residence time and the specific bound fraction of Ipl1 increase at the kinetochores during metaphase (from 3.4 ± 0.2 s to 5.7 ± 0.3 s and from 13.4 ± 1.5% to 25.5 ± 2%, Fig 3C). We have also shown that the tension across the kinetochores evicts Ipl1 from the kinetochore, consistent with the recent report (Edgerton et al, 2023). The absence of Ctf19 reduces the specific bound fraction of Ipl1 without changing the residence time significantly. This result suggests that Ctf19 is not essential for the rapid turnover of the Ipl1 at the kinetochores. However, it provides a platform for the specific binding of Ipl1 to the kinetochore. In the absence of Bub1, the specific binding of Ipl1 to the kinetochores was abolished, suggesting the absence of the Ctf19-mediated recruitment of Ipl1 to the kinetochore. Hence, neither of these pathways of Ipl1 recruitment to the kinetochores is mutually exclusive. SMIT of CPC components showed hierarchical assembly of the CPC at the kinetochore, Bir1-Nbl1 being the first to be recruited at the kinetochores, followed by the Sli15-Ipl1 complex. In agreement with these results, several reports have demonstrated Bir1-Nbl1 independent recruitment and functions of the Sli15-Ipl1 complex to the kinetochores (Campbell & Desai, 2013; Fink et al, 2017; García-Rodríguez et al, 2019). So, the observed difference in the residence times of these complexes may represent their functional differences rather than hierarchical assembly.

The method we have developed here for the SMIT of Ipl1 in *S. cerevisiae* can be used to understand the dynamics of several other mitotic kinases, phosphatases, or any other proteins in yeast and other model systems. It is in its infancy to understand the dynamic interplay between a kinase and a phosphatase for maintaining a critical phosphorylation level of their substrates. So, this method can be a valuable tool to understand such dynamic biological processes. Recent reports have demonstrated the liquid–liquid phase separation (LLPS) phenomenon for the CPC components Survivin, Borealin, and INCENP in vitro (Bryan et al, 2024; Niedzialkowska et al, 2024). Also, it has been demonstrated by live-cell single-molecule imaging that the LLPS accelerates the target-search process for transcription machinery (Kent et al, 2020). Hence, it will be interesting to address in the future whether the CPC components show LLPS behavior in live yeast. The SMIT-based assay may provide evidence for the LLPS behavior of CPC components in live cells. Understanding the dynamic regulation of AK-B/Ipl1 may open new avenues for drug development by which its recruitment dynamics can be modulated instead of a widely used approach of inhibiting its kinase activity.

### Limitations of the study

This study has two limitations. (1) Ipl1 phosphorylates several proteins at the kinetochores during metaphase (Cse4, Ndc10, Dam1, Ndc80/Hec1, Dsn1). We cannot distinguish the binding dynamics of Ipl1 for phosphorylating each of these proteins. So, the residence time and the other parameters quantified for Ipl1 dynamics at the kinetochores are averaged out for all its kinetochore substrates. (2) Because of the diffraction-limited resolution of the microscope, we cannot distinguish the three discrete sites (inner centromere, inner kinetochore, and outer kinetochore) of Ipl1 localization at the metaphase kinetochores. So, the estimation of residence time at the kinetochores during metaphase represents the residence times of all the molecules of Ipl1 localized at these three discrete sites, along with molecules present at the spindles, if any.

## Materials and Methods

### Genetic engineering of yeast strains for SMIT of Ipl1

*S. cerevisiae* strains used in this study were derived from the haploids BY4741 and BY4742, isogenic to S288C (Research Genetics/Invitrogen) (see Table S1 for genetically engineered yeast strains and Table S2 for plasmids). Standard methods were used for yeast transformation using PCR-based homologous recombination (Güldener et al, 1996). For gene deletion and C-terminal tagging of proteins, appropriate PCR fragments were amplified (see Table S3 for primers) and were integrated into the genome by homologous recombination (Longtine et al, 1998; Janke et al, 2004). All deletions and C-terminal tags were confirmed by either diagnostic PCR or observation of the localization of the appropriate fusions by fluorescence microscopy. For SMIT experiments, the *PDR5* gene, which codes for the membrane transporter protein Pdr5, was deleted to allow the HTL (JF646-HTL) retention inside the yeast cell (Ball et al, 2016). To visualize single molecules of Ipl1, the *HaloTag-TRP1* cassette was amplified from pTSK573 and transformed into the yeast cells for endogenous integration at the C terminus of *IPL1*. As a localization marker, *CloverGFP-TUB1* was integrated at the endogenous *TUB1* locus using a plasmid *pHIS3p:CloverGFP-TUB1*+*3′UTR::URA3*, digested with BsaB1 to integrate at the *TUB1* locus. For *NDC10-GFP* tagging, we used pTSK405 to amplify the *3xGFP::URA3* cassette for endogenous tagging. For auxin-inducible degradation of Cdc20 and Glc7, the endogenous copies were C-terminally fused with AID*-6HA-Hyg (Morawska & Ulrich, 2013) by amplifying the cassette from the plasmid pHyg-AID*-6HA. *pADH1-AFB2-CYCT1* cassette was integrated at the *TRP1* locus using pTSK559 (digested with SwaI).

Table S1. List of diploid yeast strains used for this study.

Table S2. List of plasmids used in this study.

Table S3. List of primers used in this study.

### Culturing cells for single-molecule imaging

The cells were streaked on the CSM plate from the glycerol stocks (from the −80°C freezer) and incubated at 30°C for 48 h. A single colony was inoculated in 5 ml CSM broth and grown at 30°C under shaking conditions (230 RPM) for 20–24 h. 50 μl of this culture was inoculated in fresh 3 ml CSM broth and grown at 30°C under shaking conditions (230 RPM) for 5–6 h to bring the cells to the log phase. 1 ml cell suspension was taken in a 14-ml round-bottom PP test tube with a snap cap; JF646-HTL was added at 30 nM concentration and kept for shaking for 30 min. Cells were pelleted by centrifugation (447*g* for 2 min) and washed twice with 3 ml of fresh prewarmed CSM media to remove unbound JF646-HTL (this step is optional, as unbound JF646-HTL will not give fluorescence). Cells were finally resuspended in 20 μl of CSM media. 3 μl of this cell suspension was taken on the LabTek II imaging chamber, covered by a nutrient agarose pad (8 × 8 mm, CSM + 2% SeaKem GTG Agarose), and the cells were then imaged for ∼1 h using a single-molecule imaging microscope. Every hour, fresh cells were taken for imaging.

To arrest the cells at metaphase (using CDC20-AID*), 1 mM auxin was added to the 5–6 h grown log phase cells and kept for shaking for an additional 2 h, followed by incubation with JF646-HTL (30 nM) for a further 30 min (Fig S2A). Cells were pelleted by centrifugation (447*g* for 2 min) and washed twice with 3 ml of fresh prewarmed CSM + 1 mM auxin media to remove unbound JF646-HTL. Cells were finally resuspended in 20 μl of CSM + 1 mM auxin media. 3 μl of this cell suspension was taken on the LabTek II imaging chamber, covered by a nutrient agarose pad (8 × 8 mm, CSM + 1 mM auxin + 2% SeaKem GTG Agarose), and the cells were then imaged for ∼1 h.

To simulate the condition for kinetochores without tension (Fig 3B, right panel), benomyl treatment (90 μg/μl) for 1 h (including the last 30-min incubation with 30 nM JF646-HTL) was given after 2 h of auxin treatment (Fig S2A). Cells were pelleted by centrifugation (447*g* for 2 min) and washed twice with 3 ml of fresh prewarmed CSM + 1 mM auxin + 90 μg/μl benomyl to remove unbound JF646-HTL. Cells were finally resuspended in 20 μl of CSM + 1 mM auxin + 90 μg/μl benomyl media. 3 μl of this cell suspension was taken on the LabTek II imaging chamber, covered by a nutrient agarose pad (8 × 8 mm, CSM + 1 mM auxin + 90 μg/μl benomyl + 2% SeaKem GTG Agarose), and the cells were then imaged for ∼1 h.

### Single-molecule imaging

Single-molecule imaging was performed on a Leica DMi8 Infinity TIRF inverted fluorescence microscope equipped with a 100X 1.47 NA oil immersion objective lens, a Photometrics Prime 95B sCMOS camera, and a 638-nm 150 mW laser (referred to as “single-molecule imaging microscope”). The microscope, camera, and lasers were controlled by Leica LASX software version 3.8.6. Cells were focused using the FITC channel under the wide-field illumination, and the best field was selected based on the fluorescence from CloverGFP-Tub1 or Ndc10-3xGFP. Single-focal plane time-lapse videos were acquired with two imaging regimes: (1) the “fast-imaging” regime acquires time-lapse videos with 15-ms time intervals (10-ms exposure time, 5-ms camera processing time, 100% laser power) to quantify the diffusive behavior of Ipl1 (Fig S2B). However, extensive photobleaching because of high laser power and continuous imaging curtails the estimation of the residence times. (2) The “slow-imaging” regime acquires time-lapse videos with 200-ms time intervals, including 50-ms exposure time, at 30% laser power (Fig S2B, Video 1 and Video 2) (Podh et al, 2022). The low laser power and 200-ms time interval allow the molecules to be visible for a long time before they photobleach. Also, the long exposure (i.e., 50 ms) allows the fast-diffusing molecules to blur out because of motion blur and selectively visualize the chromatin-bound molecules to estimate the residence times. Combining the two imaging regimes provides a holistic and quantitative view of various diffusive behaviors and kinetic subpopulations.

### Single-molecule tracking

#### Estimation of bound molecules *C*_*eq*_

The particle tracking was performed on the “slow-imaging” videos using MATLAB-based “TrackRecord” software (Mazza et al, 2013; Mehta et al, 2018; Podh et al, 2022, 2023). The software provides automated features for particle detection (puts a threshold on intensity), tracking (using the nearest neighbor algorithm with molecules allowed to move a maximum of six pixels from one frame to the next, and only tracks that are at least four frames or longer, and gaps of four frames or longer in the tracks were filled to compensate for fluorophore blinking), photobleaching correction, and quantification of residence time (survival probability distribution).

As for most of the experiments we track Ipl1 at the metaphase kinetochores, we took Ndc10-3xGFP foci in metaphase cells as a benchmark to define bound molecules at the kinetochores. Ndc10 is a bona fide kinetochore protein, and the previous report has shown strong colocalization between Ndc10-CFP and Ipl1-YFP during metaphase (Buvelot et al, 2003). We performed tracking of Ndc10-3xGFP foci in metaphase cells (from videos acquired with a slow-imaging regime) and quantified the maximum distance moved by the Ndc10-3xGFP foci using a histogram of jump distances (Fig S2C). We identify binding events for the molecule of interest by analyzing trajectory data and extracting segments where the distance moved between consecutive frames, for about 99% of the foci, is less than or equal to this maximum displacement, *r*_*min*_ (510 nm), and the end-to-end displacement for each particle is greater than a cutoff, *r*_*max*_ (730 nm).

Also, if a molecule diffuses freely but comes back to a distance less than *r*_*min*_ in consecutive frames, it should not be counted as a bound molecule. Therefore, the molecule must fulfill a minimum number of consecutive frames (*N*_*min*_) cutoff to be counted as bound (Fig S2D).

The *N*_*min*_ value used for 200-ms time interval videos was eight frames. Now, the bound fraction *C*_*eq*_ is estimated by examining all trajectories and calculating the number of frames at which the molecules were classified as bound divided by the total number of frames.

### Estimation of target-search parameter *τ*_*search*_

#### Fitting of the survival probability curve

To extract dwell time, the survival distribution, S(t), that is, *C*_*eq*_(1-*CDF*), is fitted using the method of least squares to a double-exponential decay:

$$S\left(t\right)=C_{eq\;}\left(1-CDF\right)=C_{eq}\left(F_{s}\exp\left(-tk_{s}\right)+\left(1-F_{s}\right)\;\exp\left(-tk_{ns}\right)\right),$$ (1)

where *k*_*s*_ and *k*_*ns*_ are the dissociation rates of the molecule to specific and nonspecific binding sites. Considering *τ*_*s*_/*τ*_*ns*_ to be the average dwell time for specific/nonspecific binding, *k*_*s*_ and *k*_*ns*_ are inverse of *τ*_*s*_ and *τ*_*ns*_, respectively. *F*_*s*_(1-*F*_*s*_) is the fraction of specific (nonspecific) binding molecules to total bound molecules. CDF is the cumulative distribution function of dwell time.

The bound fraction estimated by the method mentioned above underestimates the true bound fraction as only those particles that are bound for the frames more than *N*_*min*_ are counted. To address this underestimation of bound molecules and thus estimating a “true” bound fraction, extrapolation of the survival probability plot back to time t = 0 is done. That is why the S(t) curve is fitted to *C*_*eq*_(1-*CDF*).

To check for overfitting, the distribution is also fit to a single-component exponential:

$$S\left(t\right)=C_{eq}\exp\left(-kt\right).$$ (2)

The fits are compared using an *F* test to ensure that the two-component model significantly improves over the single-component decay. The values of the *F* test and associated *P*-values are shown in all the survival probability distributions (residence time analysis plots) in the main and supplementary Figures. For single-exponential fitting, the value of the *F* test is zero and the associated *P*-value is 1, whereas for double-exponential fitting, the value of the *F* test is larger and the associated *P*-value is 0.

### Average dwell time calculation

The average residence time is then estimated as follows:

$$\bar{\tau }=F_{s}\tau_{s}+\left(1-F_{s}\right)\tau_{ns}.$$ (3)

From *F*_*s*_, we can obtain the number of encounters with nonspecific binding sites before encountering a specific binding site, *N*_*trails*_ = 1/*F*_*s*_, and from *C*_*eq*_, we can get the average free time between two binding events, *τ*_3*D*_ by

$$\tau_{3D}=\;\frac{\bar{\tau }\left(1-C_{eq}\right)}{C_{eq}}.$$ (4)

So, the search time, that is, the time for locating a specific site, can be given as

$$\tau_{search}=N_{trials}\tau_{3D}+\left(N_{trials}-1\right)\tau_{ns}.$$

The differences in the residence times or fractions of specific bound molecules between two conditions were statistically checked by the Z-test to derive the *P*-values. The *P*-values are mentioned in the respective figures (Fig S2E).

### For diffusion analysis

For quantifying diffusion parameters (fraction of bound/unbound molecules and mean diffusion coefficient), particle tracking was performed using videos acquired by the “fast-imaging regime” using DiaTrack version 3.05, with the following settings as previously described (Ranjan et al, 2020; Podh et al, 2022): remove blur 0.07, remove dim 45–150, maximum jump 6 pixels, where each pixel was 110 nm. This software determines the precise position of single molecules by Gaussian intensity fitting and assembles particle trajectories over multiple frames. The trajectory data exported from DiaTrack were further converged into a single .csv file using a custom computational package, “Sojourner.”

We used SPTAnalyzer, the in-house MATLAB script for diffusion analysis. In this analysis, we have discarded tracks with at most six displacements. After filtering the data, we fitted the 30- to 75-ms range linearly to determine the diffusion coefficient (D). The D for that specific track was determined by dividing the slope of the linear fitting with R^2^ ≥ 0.8 criteria by 4. We have kept the bin width 0.26 μm. The fitting yields the fractions of the bound (*F*_*bound*_) and unbound molecules (*F*_*free*_) by fitting two Gaussians.

Quantifying diffusion parameters using MSD-based logD histograms can produce unreliable results because this method is highly dependent on the choice of track length, and linear fits to MSD may yield misleading diffusion coefficients (D values) for very stable molecules. Therefore, MSD-based analysis can be used to identify resolvable states (e.g., two or three states) within the entire population, but to accurately quantify diffusivity, the Spot-On web interface was used.

The Spot-On (Hansen et al, 2018) analysis was performed on three frames or longer trajectories. The bound fraction and mean diffusion coefficient were extracted from the CDF of observed displacements over different time intervals. The cumulative displacement histograms were fitted with a two-state model.

$$p\left(r,\tau \right)=F_{1}\;{\frac{r}{2\left(D_{1}\tau +\sigma^{2}\right)}}^{\frac{-r2}{e^{4}\left(D_{1}\tau +\sigma^{2}\right)}}+Z_{CORR}\left(\tau ,Z,D_{2}\right)F_{2}\;{\frac{r}{2\left(D_{1}\tau +\sigma^{2}\right)}}^{\frac{-r2}{e^{4}\left(D_{1}\tau +\sigma^{2}\right)}},$$

where F_1_ and F_2_ are bound and free fractions, σ is single-molecule localization error, D_1_ and D_2_ are diffusion coefficients of bound and free fractions, and Z_CORR_ is the correction factor for fast molecules moving out of the axial detection range (Hansen et al, 2018). The axial detection range for JF646 on our setup is 294 nm. The following settings were used on the Spot-On web interface: bin width 0.01, number of time points 8, jumps to consider 4, use entire trajectories – No, Max jump (μm) 1. For model fitting, the following parameters were selected: D_bound_ (μm^2^/s) min 0.0001 max 0.5, D_free_ (μm^2^/s) min 0.5 max 5, F_bound_ min 0 max 1, Localization error (μm)-Fit from data-Yes min 0.01 max 0.1, dZ (μm) 0.65 for JF646, Use Z Correction-Yes, Model Fit CDF, Iterations 3.

### Indirect immunofluorescence

Cells were fixed with 4.5% formaldehyde for 2 h at room temperature. The fixed cells were washed once with PBS and once with spheroplasting buffer (1.2 M sorbitol, 0.1 M phosphate buffer, pH 7.5) and resuspended in the same solution. Cells were spheroplasted using Zymolyase 20T in the presence of 25 mM β-mercaptoethanol for 1 h at 30°C. The spheroplasts were washed with a spheroplasting solution and transferred to poly-L-lysine–coated slides with Teflon-coated wells. The spheroplasts were flattened and permeabilized by immersing the slide in methanol for 5 min and acetone for 30 s. Blocking was done using 10 mg/ml BSA in PBS for 15 min. All the primary and secondary antibodies were diluted in the blocking reagent. Spheroplasts were incubated with primary antibodies for 1 h, washed thrice with PBS, and incubated with pre-adsorbed secondary antibodies for 1 h in the dark. After further washes with PBS, samples were incubated with a mounting medium Vectashield (Victor Laboratories) for 15 min in the dark.

Antibody dilution: rat anti-tubulin antibody (cat no. MCS78G, dilution: 1:200; Bio-Rad), TRITC-conjugated goat anti-rat antibody (cat no. 112-025-167, dilution: 1:200; Jackson ImmunoResearch).

### Protein isolation and Western blotting

The log phase culture was grown until OD_600_: 1. Cells were harvested and resuspended with 400 μl 0.1M NaOH for 30 min at room temperature. Cells were centrifuged, and the supernatant was discarded. Cell pellets were resuspended with 1X Laemmli sample buffer (2% SDS, 10% glycerol, 5% 2-mercaptoethanol, 0.002% bromophenol blue, and 60 mM Tris–HCl [pH 6.8]). An equal volume of 0.5 mm glass beads was added, and the mixture was vigorously vortexed for 2 min and incubated at 95°C for 10 min. Then, lysates were centrifuged in microfuge tubes at 18,894*g* for 10 min. Protein extracts were resolved on SDS–polyacrylamide gel, blotted onto PVDF membranes, and incubated with the following primary and secondary antibodies.

Antibody dilutions: Anti-HA rabbit mAb (cat no. 3724S, dilution: 1:2,500; Cell Signaling Technology), anti-c-Myc (9B11) mouse mAb (cat no. 2276S, dilution: 1:2,500; Cell Signaling Technology), HRP-conjugated goat anti-rabbit antibody (cat no. 111-035-003, dilution: 1:10,000; Jackson ImmunoResearch), HRP-conjugated goat anti-mouse antibody (cat no. 115-035-003, dilution: 1:10,000; Jackson ImmunoResearch).

### Spot test

10-fold serial dilutions were made from the log phase cultures of all the strains at an OD_600_ of 1. 2 μl of cell suspension from each dilution was placed on YPD agar plates (and YPD agar + 1 mM auxin plates). Plates were incubated for 48 h at 30°C, and images were taken using the gel documentation system.

## Supplementary Material

Reviewer comments

## Data Availability

The SMIT data used for this article have been deposited to the Mendeley database: Podh et al, 2025.
