# Supplementary material for: Single-molecule tracking reveals the dynamic turnover of Ipl1 at the kinetochores in Saccharomyces cerevisiae
Source: Life Sci Alliance. 2025 Apr 18;8(7):e202503290. doi: 10.26508/lsa.202503290 (PMC12008175; doi:10.26508/lsa.202503290)
Supplement: Supplementary file 6 [file LSA-2025-03290_SdataFS5.pptx]

## Slide 1
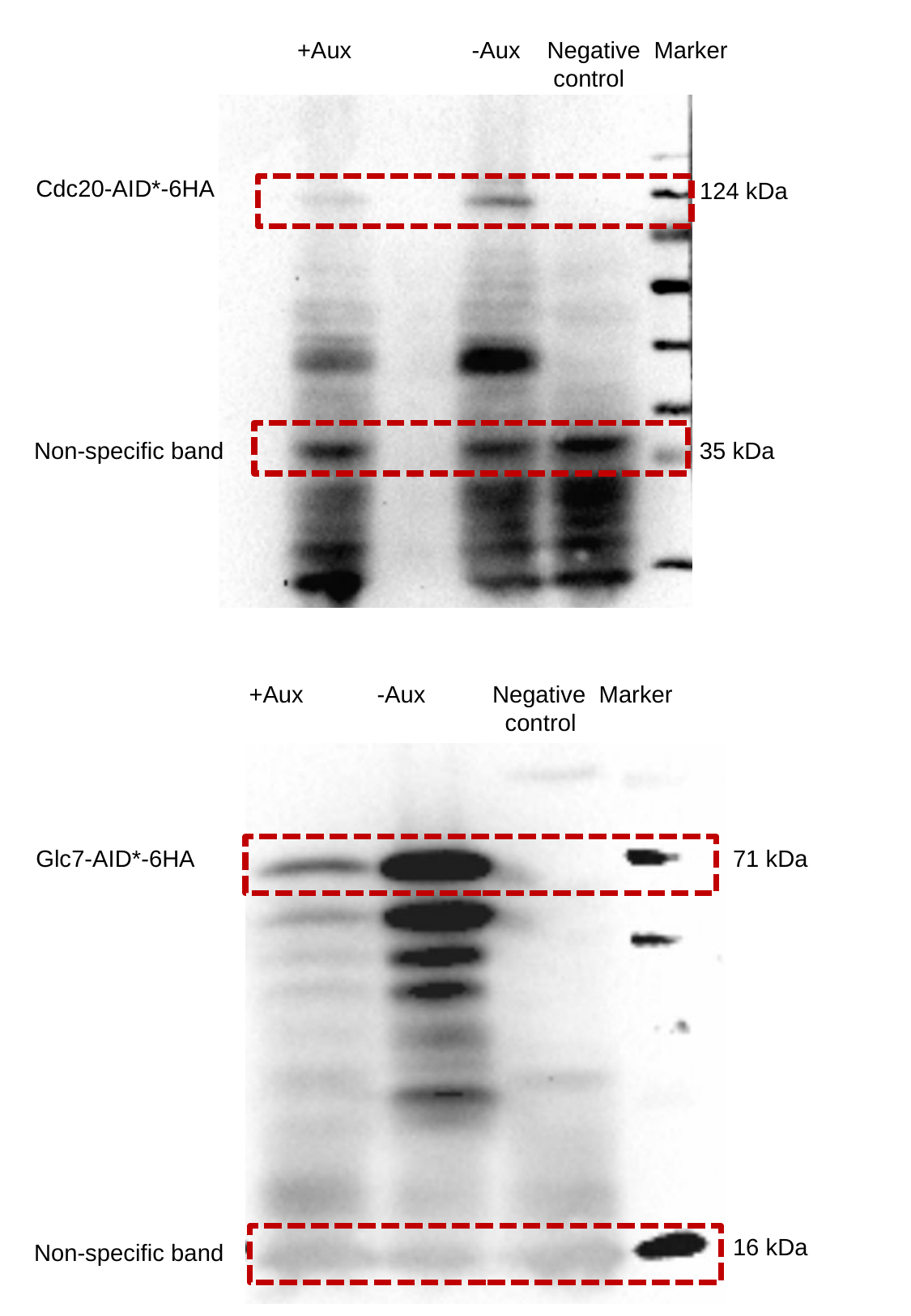

+Aux -Aux Negative Marker 		 control
Cdc20-AID*-6HA
124 kDa
Non-specific band
35 kDa
 +Aux -Aux Negative Marker 		 control
71 kDa
Glc7-AID*-6HA
16 kDa
Non-specific band
