# Supplementary material for: Single-molecule tracking reveals the dynamic turnover of Ipl1 at the kinetochores in Saccharomyces cerevisiae
Source: Life Sci Alliance. 2025 Apr 18;8(7):e202503290. doi: 10.26508/lsa.202503290 (PMC12008175; doi:10.26508/lsa.202503290)
Supplement: Supplementary file 8 [file LSA-2025-03290_TableS1.docx]

**Table S1. List of diploid yeast strains used for this study.**

All the yeast strains are derived from BY4741 (MATa) and BY4742 (MATalpha) (S288C background, Research Genetics/Invitrogen, USA).

| **Sr. No** | **Strain ID** | **Genotype** | **Mating type** | **Source** |
| --- | --- | --- | --- | --- |
|  | GMY003 | *his3-d1 leu2-d0 met15-d0 ura3-d0 pdr5Δ::LoxP trp1d::pADH-AFB2-LEU2 CDC20-AID*-9Myc-HIS3 IPL1-HaloTag-TRP1 ctf19Δ::hphNT1* | *MATa* | This study |
|  | GMY004 | *his3-d1 leu2-d0 lys2-d0 ura3-d0 pdr5Δ::LoxP CDC20-AID*-9Myc-HIS3 tub1Δ::pHIS3p:CloverGFP-TUB1+3'UTR-URA3 ctf19Δ::hphNT1* | *MATα* | This study |
|  | GMY009 | *his3-d1 leu2-d0 met15-d0 ura3-d0 pdr5Δ::LoxP trp1d::pADH-AFB2-LEU2 CDC20-AID*-9Myc-HIS3 IPL1-HaloTag-TRP1 ctf19Δ::hphNT1*  *his3-d1 leu2-d0 lys2-d0 ura3-d0 pdr5Δ::LoxP CDC20-AID*-9Myc-HIS3 tub1Δ::pHIS3p:CloverGFP-TUB1+3'UTR-URA3 ctf19Δ::hphNT1* | Diploid | This study |
|  | GMY011 | *his3-d1 leu2-d0 met15-d0 ura3-d0 pdr5Δ::LoxP trp1d::pADH-AFB2-LEU2 CDC20-AID*-9Myc-HIS3 BIR1-HaloTag-TRP1* | *MATa* | This study |
|  | GMY012 | *his3-d1 leu2-d0 met15-d0 ura3-d0 pdr5Δ::LoxP trp1d::pADH-AFB2-LEU2 CDC20-AID*-9Myc-HIS3 NBL1-HaloTag-TRP1* | *MATa* | This study |
|  | GMY013 | *his3-d1 leu2-d0 met15-d0 ura3-d0 pdr5Δ::LoxP trp1d::pADH-AFB2-LEU2 CDC20-AID*-9Myc-HIS3 BIR1-HaloTag-TRP1*  *his3-d1 leu2-d0 lys2-d0 ura3-d0 pdr5Δ::LoxP CDC20-AID*-9Myc-HIS3 tub1Δ::pHIS3p:CloverGFP-TUB1+3'UTR-URA3* | Diploid | This study |
|  | GMY014 | *his3-d1 leu2-d0 met15-d0 ura3-d0 pdr5Δ::LoxP trp1d::pADH-AFB2-LEU2 CDC20-AID*-9Myc-HIS3 NBL1-HaloTag-TRP1*  *his3-d1 leu2-d0 lys2-d0 ura3-d0 pdr5Δ::LoxP CDC20-AID*-9Myc-HIS3 tub1d::pHIS3p:CloverGFP-TUB1+3'UTR-URA3* | Diploid | This study |
|  | GMY020 | *his3-d1 leu2-d0 met15-d0 ura3-d0 pdr5Δ::LoxP trp1d::pADH-AFB2-LEU2 CDC20-AID*-9Myc-HIS3 SLI15-HaloTag-TRP1* | *MATa* | This study |
|  | GMY041 | *his3-d1 leu2-d0 met15-d0 ura3-d0 pdr5Δ::LoxP trp1d::pADH-AFB2-LEU2 CDC20-AID*-9Myc-HIS3 IPL1-HaloTag-TRP1 GLC7-AID*-6HA-hphNT1* | *MATa* | This study |
|  | GMY042 | *his3-d1 leu2-d0 lys2-d0 ura3-d0 pdr5Δ::LoxP CDC20-AID*-9Myc-HIS3 tub1d::pHIS3p:CloverGFP-TUB1+3'UTR-URA3 GLC7-AID*-6HA-hphNT1* | *MATα* | This study |
|  | GMY043 | *his3-d1 leu2-d0 met15-d0 ura3-d0 pdr5Δ::LoxP trp1d::pADH-AFB2-LEU2 CDC20-AID*-9Myc-HIS3 IPL1-HaloTag-TRP1 GLC7-AID*-6HA-hphNT1*  *his3-d1 leu2-d0 lys2-d0 ura3-d0 pdr5Δ::LoxP CDC20-AID*-9Myc-HIS3 tub1d::pHIS3p:CloverGFP-TUB1+3'UTR-URA3 GLC7-AID*-6HA-hphNT1* | Diploid | This study |
|  | GMY044 | *his3-d1 leu2-d0 met15-d0 ura3-d0 pdr5Δ::LoxP trp1d::pADH-AFB2-LEU2 CDC20-AID*-9Myc-HIS3 SLI15-HaloTag-TRP1*  *his3-d1 leu2-d0 lys2-d0 ura3-d0 pdr5Δ::LoxP CDC20-AID*-9Myc-HIS3 tub1d::pHIS3p:CloverGFP-TUB1+3'UTR-URA3* | Diploid | This study |
|  | YTK20 | *his3-d1 leu2-d0 met15-d0 ura3-d0* | *MATa* | This study |
|  | YTK21 | *his3-d1 leu2-d0 met15-d0 ura3-d0* | *MATα* | This study |
|  | YTK1500 | *his3-d1 leu2-d0 met15-d0 ura3-d0 pdr5-d::LoxP (KAN marker rescued)* | *MATa* | This study |
|  | YTK1501 | *his3-d1 leu2-d0 met15-d0 ura3-d0 pdr5-d::LoxP (KAN marker rescued)* | *MATα* | This study |
|  | GMY100 | *his3-d1 leu2-d0 met15-d0 ura3-d0 his3-d1 leu2-d0 lys2-d0 ura3-d0* | Diploid | This study |
|  | YTK1793 | *his3-d1 leu2-d0 met15-d0 ura3-d0 HHT1-HaloTag-URA3 pdr5Δ::LoxP-hphNT1-LoxP*  *his3-d1 leu2-d0 lys2-d0 ura3-d0 ace1Δ::URA3 Cu1::(LacO)256 TRP1::pHis3-GFP-FFAT-LacI-NLS- HIS3 pdr5Δ::LoxP-LEU2-LoxP* | Diploid | This study |
|  | YTK1734 | *his3-d1 leu2-d0 met15-d0 ura3-d0 pdr5Δ::LoxP trp1d::pADH-AFB2-LEU2 CDC20-AID*-9Myc-HIS3 IPL1-HaloTag-TRP1* | *MATa* | This study |
|  | YTK1800 | *his3-d1 leu2-d0 lys2-d0 ura3-d0 pdr5Δ::LoxP CDC20-AID*-9Myc-HIS3 tub1d::pHIS3p-CloverGFP-TUB1+3'UTR-URA3* | *MATα* | This study |
|  | YTK1803 | *his3-d1 leu2-d0 lys2-d0 ura3-d0 pdr5Δ::LoxP CDC20-AID*-9Myc-HIS3 NDC10-3xGFP-URA3* | *MATα* | This study |
|  | YTK1804 | *his3-d1 leu2-d0 lys2-d0 ura3-d0 pdr5Δ::LoxP CDC20-AID*-9Myc-HIS3 tub1d::pHIS3p-CloverGFP-TUB1+3'UTR-URA3*  *his3-d1 leu2-d0 met15-d0 ura3-d0 pdr5Δ::LoxP trp1d::pADH-AFB2-LEU2 CDC20-AID*-9Myc-HIS3 IPL1-HaloTag-TRP1* | Diploid | This study |
|  | YTK1807 | *his3-d1 leu2-d0 met15-d0 ura3-d0 pdr5Δ::LoxP trp1d::pADH-AFB2-LEU2 CDC20-AID*-9Myc-HIS3 IPL1-HaloTag-TRP1*  *his3-d1 leu2-d0 lys2-d0 ura3-d0 pdr5Δ::LoxP CDC20-AID*-9Myc-HIS3 NDC10-3xGFP-URA3* | Diploid | This study |
|  | GMY301 | *his3-d1 leu2-d0 lys2-d0 ura3-d0 pdr5-d::LoxP CDC20-AID*-9Myc::HIS3 tub1d::pHIS3p:CloverGFP-TUB1+3'UTR::URA bub1-d::KANMX* | *MATα* | This study |
|  | GMY302 | *his3-d1 leu2-d0 met15-d0 ura3-d0 pdr5-d::LoxP trp1d::pADH-AFB2::LEU2 CDC20-AID*-9Myc::HIS3 IPL1-HaloTag::TRP1 bub1-d::NATMX* | *MATa* | This study |
|  | GMY303 | *his3-d1 leu2-d0 lys2-d0 ura3-d0 pdr5-d::LoxP CDC20-AID*-9Myc::HIS3 tub1d::pHIS3p:CloverGFP-TUB1+3'UTR::URA bub1-d::KANMX*  *his3-d1 leu2-d0 met15-d0 ura3-d0 pdr5-d::LoxP trp1d::pADH-AFB2::LEU2 CDC20-AID*-9Myc::HIS3 IPL1-HaloTag::TRP1 bub1-d::NATMX* | Diploid | This study |
|  | SBY2189 | *bar1 ura3-1 leu2-3,112 his3-11 trp1-1 can1-100 ade2-1 ipl1-321 pHIS3p* | *MATa* | Kotwaliwale et al (2007^)^ |
|  | GMY409 | *bar1 ura3-1 leu2-3,112 his3-11 trp1-1 can1-100 ade2-1 ipl1-321 pHIS3p:CloverGFP-TUB1+3'UTR::URA3:: IPL1-NewHalo::TRP1* | *MATa* | This study |
