# Supplementary material for: Single-molecule tracking reveals the dynamic turnover of Ipl1 at the kinetochores in Saccharomyces cerevisiae
Source: Life Sci Alliance. 2025 Apr 18;8(7):e202503290. doi: 10.26508/lsa.202503290 (PMC12008175; doi:10.26508/lsa.202503290)
Supplement: Supplementary file 9 [file LSA-2025-03290_TableS2.docx]

**Table S2. List of plasmids used in this study.**

| **Sr No.** | **Plasmid name** | **Description** | **Source** |
| --- | --- | --- | --- |
| 1 | pTSK573 | For C-terminal fusion of the *HaloTag* using *TRP1* selection marker | [Addgene: 190881](https://www.addgene.org/190881/) |
| 2 | *pHyg-AID*-6HA* | For C-terminal fusion of the *AID* (Auxin-Inducible Degron) tag for the conditional depletion of a protein of interest gene, selection marker: Hygromycin B | [Addgene: 99520](https://www.addgene.org/99520/) |
| 3 | pAG32 | For gene deletion using hygromycin B selection marker | [Euroscarf: P30106](http://www.euroscarf.de/plasmid_details.php?accno=P30106) |
| 4 | *pHIS3p:CloverGFP-TUB1+3’UTR::URA3* | For the expression of CloverGFP-Tub1 as a localization marker | [Addgene: 50636](https://www.addgene.org/50636/) |
| 5 | pTSK559 | For the expression of *AFB2* from *ADH1* promoter | Mehta et al (2018) |
| 6 | pTSK405 | For C-terminal fusion with *3xGFP* tag (*NDC10-3xGFP*, *IPL1-3xGFP*) | Mehta et al (2018) |
