# Supplementary material for: Single-molecule tracking reveals the dynamic turnover of Ipl1 at the kinetochores in Saccharomyces cerevisiae
Source: Life Sci Alliance. 2025 Apr 18;8(7):e202503290. doi: 10.26508/lsa.202503290 (PMC12008175; doi:10.26508/lsa.202503290)
Supplement: Supplementary file 10 [file LSA-2025-03290_TableS3.docx]

**Table S3. List of primers used in this study.**

Homologous sequences for site-specific integration/recombination are shown in blue fonts. Sequences homologous to plasmid DNA and genomic DNA are shown in red and black fonts, respectively.

| **Sr No.** | **Primer Id** | **Description** | **Template** | **Sequence** |
| --- | --- | --- | --- | --- |
| 1 | GM29 | Forward primer for *SLI15-HaloTag-TRP1* | pTSK573 | *ATCCTAGGCTAAACAGGTTGAAACCGCGTCAAATTGTGCCCAAAAGGTCTCAAGCGGCCGCCGCTGCTGC* |
| 2 | GM30 | Reverse primer for *SLI15-HaloTag-TRP1* | pTSK573 | *ATTTAATGTTAACCAGTTTGAATTTTTCTTTTCTGGGGTAATCGAATTCACTATTTCTTAGCATTTTTGACG* |
| 3 | GM31 | Diagnostic forward primer for *SLI15-HaloTag-TRP1* | *S. cerevisiae* genomic DNA | *GGCTAGCGTAACTTTAGCGG* |
| 4 | GM32 | Diagnostic reverse primer for *SLI15-HaloTag-TRP1* | *S. cerevisiae* genomic DNA | *CGTAATGCAGGGGGAATACC* |
| 5 | GM33 | Forward primer for *BIR1-HaloTag-TRP1* | pTSK573 | *TTTGGAAGATGACAATCAATTGATCGATATTGCTAAGAAAATGGGCATTTTACAAGCGGCCGCCGCTGCTGC* |
| 6 | GM34 | Reverse primer for *BIR1-HaloTag-TRP1* | pTSK573 | *AAACTACAAAAAATACAAACCTTTAGCCTGTTTATCAAATTAGTTAGCTACTATTTCTTAGCATTTTTGACG* |
| 7 | GM35 | Diagnostic forward primer for *BIR1-HaloTag-TRP1* | *S. cerevisiae* genomic DNA | *GCAGAAGAGTTGGACATGAC* |
| 8 | GM36 | Diagnostic reverse primer for *BIR1-HaloTag-TRP1* | *S. cerevisiae* DNA | *CAGATATCTGCGATGCGGCG* |
| 9 | GM37 | Forward primer for *NBL1-HaloTag-TRP1* | pTSK573 | *CAAAGGAACTAATCAGAGAGGTGCTTGAACAGGAAGGACGCCGTATAGAACAAGCGGCCGCCGCTGCTGC* |
| 10 | GM38 | Reverse primer for *NBL1-HaloTag-TRP1* | pTSK573 | *AGGTGCATCATTGCGAATACCGAGAAAGGGTCCATTATACGAACTAATCACTATTTCTTAGCATTTTTGACG* |
| 11 | GM39 | Diagnostic forward primer for *NBL1-HaloTag-TRP1* | *S. cerevisiae* genomic DNA | *CTCTACCCCCAACCTTCACC* |
| 12 | GM40 | Diagnostic reverse primer for *NBL1-HaloTag-TRP1* | *S. cerevisiae* genomic DNA | *GGGATGTAACCACACGCTGC* |
| 13 | GM41 | Forward primer for *GLC7-AID*-6HA-hphNT1* | *pHyg-AID*-6HA* | *AGCCAGCCCAAAAAAGTCTACCAAGGCAAGCTGGGGGTAGAAAGAAAAAACGTACGCTGCAGGTCGAC* |
| 14 | GM42 | Reverse primer for *GLC7-AID*-6HA-hphNT1* | *pHyg-AID*-6HA* | *TAATAAGTATTTTCCTTTTTAAACTTTGATTTAGGACGTGAATCTATTTAATCGATGAATTCGAGCTCG* |
| 15 | GM43 | Diagnostic forward primer for *GLC7-AID*-6HA-hphNT1* | *S. cerevisiae* genomic DNA | *CGCTGGTGCAATGATGAGTG* |
| 16 | GM79 | Diagnostic reverse primer for *GLC7-AID*-6HA-hphNT1* | *S. cerevisiae* genomic DNA | *GACGAGTGATGATTGCATCTTCC* |
| 17 | GM49 | Forward primer for *ctf19*Δ | pAG32 | *CTTGGAGCTAGTGTGATCTTGTTGATACTAGGTCGGCAAAGAACGCAAATGCCAGCTGAAGCTTCGTACG* |
| 18 | GM50 | Reverse primer for *ctf19*Δ | pAG32 | *TATCGGAATCGTTTAAGCAAGCCGTCCAGTTGGCAATGGCAAATGGAACAGGCCACTAGTGGATCTG* |
| 19 | GM51 | Diagnostic forward primer for *ctf19*Δ | *S. cerevisiae* genomic DNA | *GGTAATGTAACCGGTAATGG* |
| 20 | GM52 | Diagnostic reverse primer for *ctf19*Δ | *S. cerevisiae* genomic DNA | *CGACGATGCAAATGAATTGC* |
| 21 | GM66 | Forward primer for *pdr5*Δ | pAG32 | *AGACCCTTTTAAGTTTTCGTATCCGCTCGTTCGAAAGACTTTAGACAAAAGCCAGCTGAAGCTTCGTACG* |
| 22 | GM67 | Reverse primer for *pdr5*Δ | pAG32 | *AAATTCAAGAAAATTGAAATGTAGAAAGCTCGCTGAATTAAGAAAAAAAAGGCCACTAGTGGATCTG* |
| 23 | GM68 | Diagnostic forward primer for *pdr5*Δ | *S. cerevisiae* genomic DNA | *CTCTTCTACGCCGTGGTACG* |
| 24 | GM69 | Diagnostic reverse primer for *pdr5*Δ | *S. cerevisiae* genomic DNA | *GAAGACGGTTCGCCATTCG* |
| 25 | GM70 | Forward primer for *IPL1-HaloTag-TRP1* | pTSK573 | *TGCATCCTTGGATACTAAGAAACATGCCCTTTTGGGAAAATAAGCGGTTACAAGCGGCCGCCGCTGCTGC* |
| 26 | GM71 | Reverse primer for *IPL1-HaloTag-TRP1* | pTSK573 | *GGGAGTGATTAATAGTGCCCTTCAAACGATTCTGTCATACTTTAATTCTACTATTTCTTAGCATTTTTGACG* |
| 27 | GM72 | Diagnostic forward primers for *IPL1-HaloTag-TRP1* | *S. cerevisiae* genomic DNA | *GATAGAATGCGCCTTGGAGAC* |
| 28 | GM73 | Diagnostic reverse primer for *IPL1-HaloTag-TRP1* | *S. cerevisiae* genomic DNA | *CTGCGAATGCTCGTCTTGAG* |
| 29 | GM144 | Forward primer for *GLC7-HaloTag-TRP1* | pTSK573 | *AGCCAGCCCAAAAAAGTCTACCAAGGCAAGCTGGGGGTAGAAAGAAAAAACAAGCGGCCGCCGCTGCTGC* |
| 30 | GM145 | Reverse primer for *GLC7-HaloTag-TRP1* | pTSK573 | *TAATAAGTATTTTCCTTTTTAAACTTTGATTTAGGACGTGAATCTATTTACTATTTCTTAGCATTTTTGACG* |
| 31 | GM296 | Forward primer for *bub1*Δ | pAG32 | *GAAAGATTATTGACGGTTCCTATTGTTTGAATGTTAACGCTGACCAGGAAGCCAGCTGAAGCTTCGTACG* |
| 32 | GM297 | Reverse primer for *bub1*Δ | pAG32 | *GCAGGACACCAAAAAGTCACCTATGCGGGAGATGAAGGCATATTTATTCAGGCCACTAGTGGATCTG* |
| 33 | GM298 | Diagnostic forward primer for *bub1*Δ | *S. cerevisiae* genomic DNA | *GACGGTTCCTATTGTTTG* |
| 34 | GM299 | Diagnostic reverse primer for *bub1*Δ | *S. cerevisiae* genomic DNA | *GTGTTGTCATTGCTATGG* |
| 35 | GM1001 | Forward primer for *NDC10-3xGFP* tagging | pTSK405 | *GGCATGACCATCAAAATTCATTTGATGGTCTGTTAGTATATCTATCTAACCAAGCGGCCGCCGCTGCTGC* |
| 36 | GM1002 | Reverse primer for *NDC10-3xGFP* tagging | pTSK405 | *TACATGTCGGTATCCCTATACGAAACAGTTTAAACTTCGAAGCTCCCTCA GTTACTTGGTTCTGGCGAGG* |
| 37 | GM1003 | Forward primer for *IPL1-3xGFP* tagging | pTSK405 | *TGCATCCTTGGATACTAAGAAACAAGCCCTTTTGGGAAAATAAGCGGTTACAAGCGGCCGCCGCTGCTGC* |
| 38 | GM1004 | Reverse primer for *IPL1-3xGFP* tagging | pTSK405 | *GGGAGTGATTAATAGTGCCCTTCAAACGATTCTGTCATACTTTAATTCTAGTTACTTGGTTCTGGCGAGG* |
